# Positive Predictive Value of Panoramic Radiography for Assessment of the Relationship of Impacted Mandibular Third Molars with the Mandibular Canal Based on Cone-Beam Computed Tomography: A Cross-Sectional Study

**DOI:** 10.3390/diagnostics11091578

**Published:** 2021-08-31

**Authors:** Maryam Tofangchiha, Soheil Koushaei, Maryam Mortazavi, Zahra Souri, Ahad Alizadeh, Romeo Patini

**Affiliations:** 1 Department of Oral and Maxillofacial Radiology, Dental Caries Prevention Research Center, Qazvin University of Medical Sciences, Qazvin 34199-15315, Iran; mt_tofangchiha@yahoo.com; 2 Department of Oral and Maxillofacial Surgery, Dental Faculty, Qazvin University of Medical Sciences, Qazvin 34199-15315, Iran; Drkoushaei@yahoo.com; 3 Student Research Committee, Qazvin University of Medical Sciences, Qazvin 34199-15315, Iran; maryam_mortazavi85@yahoo.com (M.M.); matinsouri137265@gmail.com (Z.S.); 4 Medical Microbiology Research Center, Qazvin University of Medical Sciences, Qazvin 34199-15315, Iran; 5 Department of Head, Neck and Sense Organs, School of Dentistry, Catholic University of Sacred Heart, 00135 Rome, Italy; romeo.patini@unicatt.it

**Keywords:** radiography, panoramic, molar, third, cone-beam computed tomography

## Abstract

The aim of the present study was to assess the positive predictive value (PPV) of panoramic radiographic signs in the assessment of the relationship between impacted mandibular third molars (IMTMs) and the mandibular canal (MC). This cross-sectional study was conducted by reviewing 102 cone-beam computed tomography (CBCT) and panoramic radiographs of patients with IMTMs and radiographic signs of the contact of the IMTMs with the MC on panoramic radiographs (i.e., root apex darkening and interference with the white line). A positive relationship of the IMTM roots with the MC based on CBCT findings was recorded as the gold standard. The PPV of panoramic radiographic signs was calculated for the detection of the relationship of the IMTM root with the MC. The IMTMs were in contact with the MC on CBCT scans in 90.1% of the cases. The PPV of root apex darkening and the interference with the white line was found to be 89.09% (95% CI: (77.75, 95.88)) and 91.48% (95% CI: (79.62, 97.63)), respectively. The MC had a buccal position in 63.7%, and a lingual position in 35.2%, of the cases. The contact of IMTMs with the MC was more commonly seen in patients with a lingual position (100% of the samples). The IMTM root apex darkening and interference with the white line of the MC on panoramic radiographs had a high PPV for determination of the contact of IMTMs with the MC. Thus, presence of the above-mentioned risk factors indicates the need for subsequent 3D radiographic assessments.

## 1. Introduction

Extraction of impacted mandibular third molars (IMTMs) is among the most common oral surgical procedures performed by dental clinicians and oral and maxillofacial surgeons [1]. However, the extraction of IMTMs can cause dysesthesia of the inferior alveolar nerve (IAN), which is a serious postoperative complication [1,2]. The proximity of the mandibular canal (MC) to the IMTM root is the most important factor responsible for IAN damage. Dental radiography is a suggested diagnostic modality to prevent such complications [2]. Radiography can provide valuable information regarding the size, shape, and branching of the MC; orientation of the IMTM root (s); proximity to the adjacent anatomical structures; and complications related to third molar impaction [3]. Therefore, preoperative radiography is imperative to enhance the process of tooth extraction and to estimate and minimize the risk of IAN damage [4]. Evidence of the contact of the IMTM root and the MC on panoramic radiographs can serve as a risk factor for IMTM surgery and postoperative complications. Although panoramic radiography does not have an adequate accuracy for the assessment of this relationship, it is often requested as a primary diagnostic tool due to its availability and low cost. Thus, the risk factors for the contact of the MC with the IMTMs on panoramic radiographs should be recognized for more accurate diagnosis, and more advanced imaging modalities should be requested in case of the presence of risk factors.

Cone-beam computed tomography (CBCT) has several applications in dentistry, since it provides a 3D image of the respective area and visualizes the anatomical structures more clearly in comparison with conventional radiography. However, due to having a higher patient radiation dose than conventional imaging techniques, it should be prescribed as a complementary diagnostic tool in special cases [5]. The use of CBCT is on the rise, especially in orthodontics [6], for orthognathic surgery [7], and for implant treatments [8]. CBCT is increasingly replacing CT in dental fields, because it provides high-quality images with a lower patient radiation dose [9] and also has a lower cost [10].

**Figure 1:**
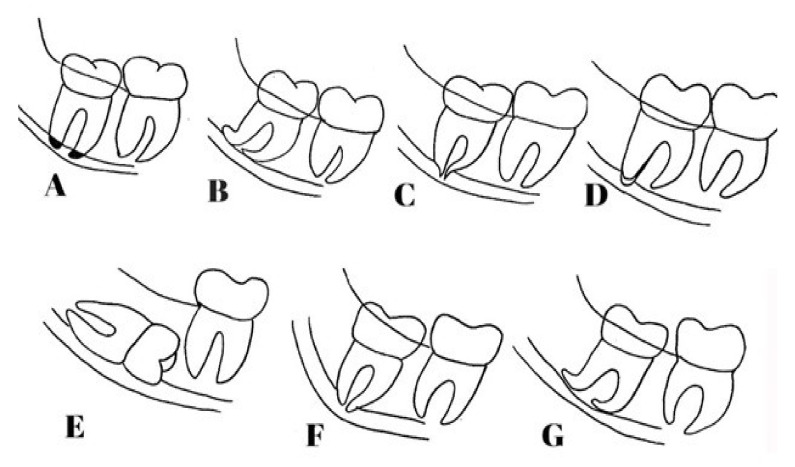
Seven radiographic signs for the contact of the IMTMs with the MC on panoramic images, based on the classification by Rood and Shehab: (**A**) root darkening, (**B**) root deflection, (**C**) root narrowing, (**D**) darkening and branching of the root, (**E**) interference with the white line, (**F**) MC diversion, and (**G**) MC narrowing.

There are seven radiographic signs that are regarded as risk factors for the contact of the IMTMs with the MC on panoramic images, according to the classification by Rood and Shehab: namely (I) root darkening, (II) root deflection, (III) root narrowing, (IV) darkening and branching of the root, (V) interference with the white line, (VI) MC diversion, and (VII) MC narrowing [11] (Figure 1). Evidence shows that interference with the white line of the MC is the most common panoramic radiographic finding, which indicates direct contact of the two structures and is associated with a high risk of nerve damage and paresthesia [12]. Moreover, root darkening is among the strongest signs for the prediction of IAN exposure and paresthesia [13].

Considering the significance of the interference with the white line and root darkening, and the risk of permanent IAN damage in the process of IMTM extraction and subsequent complications, this study aimed to address the two above-mentioned risk factors. This study sought to validate the positive predictive value (PPV) of panoramic radiographic findings for the evaluation of the relationship of the IMTMs with the MC in comparison with CBCT (as the gold standard), in order to analyze the reliability of panoramic radiography for the correct assessment of this relationship.

In addition, studies have shown that one of the factors influencing the risk of IAN damage is the relationship between the IMTM root and the MC [14,15]. Thus, in this study, the position of these structures relative to each other was reported based on CBCT data.

## 2. Methods

### 2.1. Study Design

This cross-sectional study evaluated the panoramic radiographs and CBCT scans of patients retrieved from the archives of an oral and maxillofacial radiology clinic in Qazvin, Iran, after obtaining ethical approval from the ethics committee of the university. The radiographs of patients presenting to the clinic within a one-year period were selected by census method after applying the eligibility criteria. The panoramic radiographs and CBCT scans had been requested by oral and maxillofacial surgeons for assessment of the relationship of the IMTMs with the MC. All panoramic radiographs were obtained with a Cranex D X-ray system (Soredex, Helsinki, Finland) and all CBCT scans were made with a ProMax 3D CBCT scanner (Planmeca, Helsinki, Finland) with 0.15 × 0.15 mm voxel size and 8 × 8 cm field of view.

### 2.2. Sample Size

In this study, the sample size was calculated as 102, to estimate a 95% confidence interval based on the effect size of 0.089 and PPV of 0.90 using a formula for estimating the confidence interval of a proportion. This sample size was determined for the primary outcome (PPV).

### 2.3. Evaluation of Images

Two radiologists with more than 10 years of experience participated in this study as the observers. The first radiologist selected and evaluated the panoramic radiographs exclusively, while the second radiologist examined the CBCT scans of the same patients, whose panoramic radiographs were evaluated.

**Figure 2:**
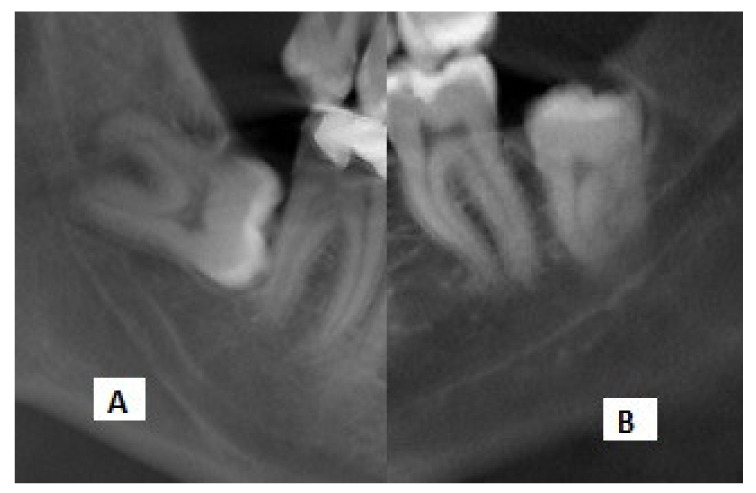
Cropped panoramic radiographs showing the (**A**) interference with the white line and (**B**) darkening of roots.

A total of 132 IMTMs with one of the risk factors of root darkening or interference with the white line of the MC on panoramic radiographs, according to the classification by Rood and Shehab, were evaluated. The radiographs were randomly selected by an experienced radiologist from the archives after applying the eligibility criteria. Information regarding the relationship of the IMTMs and the MC was recorded in a datasheet (Figure 2). Next, an experienced oral and maxillofacial radiologist, familiarized with CBCT interpretation, evaluated the cross-sectional, axial, and coronal views of the selected CBCT scans by Romexis software (R version 3.8.3, Planmeca, Helsinki, Finland) on a 19-inch monitor (Samsung, Seoul, Korea) in a semi-dark room.

**Figure 3:**
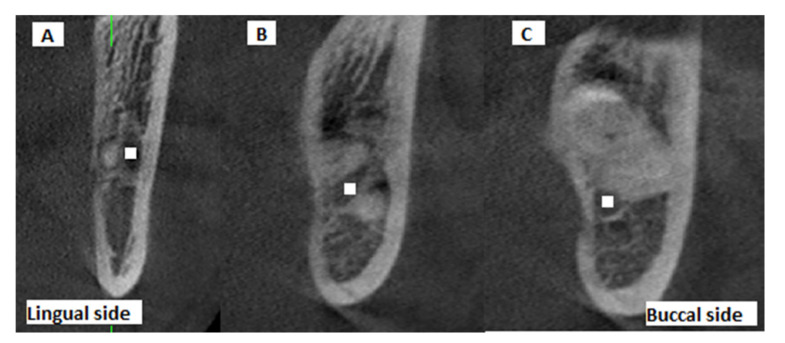
Cross-sectional CBCT views showing the buccolingual position of the IMTM apex relative to the MC: (**A**) buccal, (**B**) inter-radicular, (**C**) lingual (white squares: center of mandibular canal).

Moreover, the position of the MC relative to the root apex of the IMTM (lingual, inter-radicular or buccal position) was determined on the CBCT scans and recorded (Figure 3). The presence/absence of contact between the MC and the IMTM root was also evaluated on coronal, axial, and cross-sectional CBCT views and recorded.

The relationship between the IMTM root and the MC was determined on the CBCT scans and recorded as follows: actual contact between the MC and the IMTM root (interference with the internal space of the MC or not, observing the lower cortical border of the MC), and the buccolingual position of the MC relative to the IMTM root [15]. The CBCT data served as the gold standard.

### 2.4. Inclusion Criteria

– Samples with IMTMs visible on panoramic radiographs and CBCT scans.
– Radiographs of IMTMs showing root darkening or interference with the white line of the MC as risk factors.
– Absence of developmental and pathological lesions in the mandibular trunk due to possible change in the position of the MC in the area.
– Maturity of the apex of the IMTM root on the panoramic radiograph.

### 2.5. Exclusion Criteria

Poor quality of CBCT scans, open apices of IMTMs, and inability to detect the external borders of the MC.

### 2.6. Outcomes

The primary outcome was the PPV of the panoramic radiographic signs for assessment of the relationship of IMTMs with the MC. PPV is the proportion of the positive results in statistics and diagnostic tests that are true positive.

The secondary outcome was the frequency of contact of the IMTMs with the MC on CBCT scans.

The tertiary outcomes were the frequency of MC position relative to the root apex of the IMTM (lingual, inter-radicular, or buccal) and its correlation with gender.

### 2.7. Statistical Analysis

Data were analyzed by a statistician not involved in the radiographic assessments, using R software version 4.1.0 (R Foundation for Statistical Computing, Vienna, Austria). The frequency and percentage values were reported to describe the qualitative variables. The 95% confidence interval of the PPV was calculated using a procedure first described by Clopper and Pearson [16]. Logistic regression was applied to calculate the odds ratio and the *p*-values. *p*-values < 0.05 were considered statistically significant.

## 3. Results

A total of 132 IMTMs were initially selected; out of which, 30 (22.72%) were excluded from the study due to open apices of the IMTMs or the inability to detect the external borders of the MC. Of the remaining 102, 46 (44.6%) belonged to males and 56 (55.4%) belonged to females. Gender had no significant correlation with the contact of the IMTM root with the MC (*p* = 0.74, Table 1).

The MC had a buccal position relative to the IMTM root in 65 cases (63.7%), a lingual position in 36 cases (35.2%), and an inter-radicular position in 1 (0.1%) case.

The MC was in contact with the IMTM root in only 55 (84.6%) cases with a buccal position. However, this contact was reported in 100% of the cases with a lingual position. The logistic regression test showed that the two panoramic radiographic signs evaluated in this study reliably revealed the contact of IMTMs and the MC, as confirmed by CBCT, and that the frequency of contact was higher in the lingual position of the MC when compared with the buccal position (*p* = 0.013). No significant difference was noted between the buccal and inter-radicular (*p* = 0.67) or lingual and inter-radicular (*p* = 0.86) positions of the MC in this respect.

The IMTM root was in contact with the MC in 90.19% (95% CI: (82.70, 95.19)) of the cases, as noted on CBCT scans.

In assessment of the PPV of the root darkening radiographic sign, the results showed the agreement of this panoramic radiographic sign and the CBCT findings in 89.09% (95% CI: (77.75, 95.88)) of the cases (the IMTM root was in contact with the MC; Table 2).

As shown in Table 2, assessment of the PPV of the interference with the white line on panoramic radiographs revealed an agreement between this panoramic radiographic sign and CBCT findings in 91.48% (95% CI: (79.62, 97.63)) of the cases (the IMTM was in contact with the MC).

## 4. Discussion

Preoperative CBCT is often recommended, to minimize the risk of damage to the IAN and artery [17]. However, panoramic radiography is the most commonly used imaging modality for this purpose, due to its easy availability, lower patient radiation dose, and lower cost [18]. On the other hand, Petersen et al. showed that preoperative CBCT could not decrease the risk of neurosensory complications after surgery [19].

In this study, the frequency of contact of the IMTM root with the MC was higher when the MC had a lingual position relative to the IMTM root. The PPV of root apex darkening and interference with the white line on panoramic radiographs was found to be 89.09% and 91.48%, respectively, when compared with CBCT as the gold standard. According to the present results, the MC was in contact with the IMTM in 90.19% of the cases (92 out of 102). In 2020, Lacerda-Santos et al. [20] evaluated the relationship of the IMTMs with the MC by using CBCT and reported the presence of contact between the two in 63.5% of the cases. The present results showed that darkening of the root on panoramic radiographs, which was noted in 89% of the cases (49 out of 55), and interference with the white line of the MC on panoramic radiographs, which was noted in 91.5% of the cases (43 out of 47), were in agreement with the CBCT findings. Bell et al. [21] evaluated the agreement between panoramic radiography and CBCT findings, regarding the relationship of the IMTMs with the MC. They concluded that root darkening and interference with the white line of the MC were both effective in revealing a high risk of contact of IMTM root with the MC. This finding highlighted the need for 3D radiographic assessment, which was in line with the present results. Elkhateeb et al., [22] in 2018, evaluated the diagnostic accuracy of panoramic radiography and CBCT for prediction of the contact of IMTMs with the MC. They assessed 210 IMTMs and concluded that root darkening and interference with the white line of the MC are two highly prevalent radiographic signs on panoramic radiographs. They explained that detection of root darkening and interference with the white line on panoramic images indicates an increased possibility of contact between the IMTM and the MC, which was in agreement with the present results. The present study showed 89% and 91.5% agreement between the detection of root darkening and interference with the white line on panoramic radiographs, respectively, and the CBCT findings regarding the contact of the IMTM root and the MC.

Leung et al. [18] showed that root darkening was the only panoramic radiographic sign with a significant association with IAN damage after surgery.

Furthermore, Tantanapornkul et al. [23] showed that the observation of interference with the MC wall, alone or in combination with root darkening on panoramic radiographs, can reliably predict a positive relationship between the MC and the IMTM root. Such findings are in accordance with the present results since an agreement was observed with CBCT findings in 89% of the cases with root darkening on panoramic radiographs. The results of this study regarding the position of the MC relative to the IMTM root (buccal position in 63.7% of the cases) were in line with the findings of Yamada et al. [24] on 112 teeth, Nakamori et al. [25] on 695 teeth, Meagawa et al. [1] on 47 teeth, Miller et al. [2] on 31 teeth, Arora et al. [26] on 49 teeth, and De-Azevedo-Vaz et al. [27] on 173 mandibular third molar roots. It should be noted that all the above-mentioned studies were conducted on Asian populations. Conversely, Ghaeminia et al. [28], in their study on 40 patients (53 IMTMs); De Melo et al. [29], in their study on 29 teeth; Ohman et al. [30], in their study on 90 teeth; Monaco et al. [31], in their study on 23 teeth; and Shujaat et al. [32], in their study on 100 teeth confirmed that the MC had a lingual position relative to the IMTM root in the majority of the cases, according to CBCT findings. The above-mentioned studies were all conducted on European and South American populations. Since the results of studies conducted on Asian populations were in accordance with our findings, it appears that race may play a role in this respect.

According to the present results, the MC was in contact with the IMTM apex on the CBCT in 100% of the cases that showed lingual position of the MC relative to the IMTM root; this value was significantly higher than in cases with a buccal position of the MC (*p* = 0.013). In 2017, Kundoor et al. [33] evaluated 60 IMTMs of 40 patients with risk factors for IAN damage on their panoramic and CBCT images. They showed that the MC had a lingual position relative to the apex of the IMTM in 60% of the cases. They found that among 19 cases that showed a lingual position of the MC, the IMTM was in contact with the MC on CBCT scans in 16 cases (84.2%), which was in line with the present results. Moreover, among 36 cases that showed a buccal position of the MC, the IMTM was in contact with the MC in 15 cases (25%). In 2012, Jung et al. [34] evaluated the relationship of the IMTMs and the MC and the related factors using CBCT. They found that of 74 cases with a lingual position of the MC, 73 (98.6%) showed actual contact of the IMTM and the MC on CBCT scans, which was very close to the value obtained in the present study. In 2020, Lacerda-Santos et al. [20] assessed 2639 teeth and reported that contact of the IMTMs with the MC was more prevalent in females. Although the effect of gender on the contact of IMTMs with the MC was not significant in the present study, it was slightly more prevalent in females (*n* = 51) than males (*n* = 41).

This study had a larger sample size than previous studies on this topic [1,2,27], which increases the reliability of the results. In addition, since this study evaluated imaging modalities, and the results were not dependent on demographic factors, the results can be generalized to the entire population.

The main limitation of this study was the evaluation of only two signs of interference of the IMTM root and the MC, due to the insufficient number of other radiographic signs on the radiographs retrieved from the archives.

## 5. Conclusions

The results of the present study supported the findings of previous studies on this topic, indicating that the two panoramic radiographic signs evaluated in this study can reliably predict the contact of IMTMs and the MC, as confirmed by CBCT, and that the frequency of contact was higher in the lingual position of the MC.

## Figures and Tables

**Table 1:** Correlation of gender with the contact of the IMTM root with the MC.

Gender	Presence of Contact Number (%)	Absence of Contact Number (%)	OR (95% CI)	*p* Value
Male	41 (44.6)	5 (50)	0.804 (0.22, 2.97)	0.74
Female	51 (55.4)	5 (50)		

CI: Confidence Interval.

**Table 2:** Frequency distribution of panoramic radiographic signs and their PPV in comparison with the gold standard (CBCT)

Radiographic Sign	Presence (%)	Absence (%)	PPV (95% CI)
Root apex darkening	49 (89.09)	6 (10.91)	89.09 (77.75, 95.88)
Interference with the white line	43 (91.48)	4 (8.52)	91.48 (79.62, 97.63)
Panoramic radiographic signs (total)	92 (90.19)	10 (9.81)	90.19 (82.70, 95.19)

CI: Confidence Interval.

## Data Availability

Not applicable.
